# Neuraldecipher – reverse-engineering extended-connectivity fingerprints (ECFPs) to their molecular structures†

**DOI:** 10.1039/d0sc03115a

**Published:** 2020-09-11

**Authors:** Tuan Le, Robin Winter, Frank Noé, Djork-Arné Clevert

**Affiliations:** Department of Digital Technologies, Bayer AG Berlin Germany tuan.le2@bayer.com djork-arne.clevert@bayer.com; Department of Mathematics and Computer Science, Freie Universität Berlin Berlin Germany

## Abstract

Protecting molecular structures from disclosure against external parties is of great relevance for industrial and private associations, such as pharmaceutical companies. Within the framework of external collaborations, it is common to exchange datasets by encoding the molecular structures into descriptors. Molecular fingerprints such as the extended-connectivity fingerprints (ECFPs) are frequently used for such an exchange, because they typically perform well on quantitative structure–activity relationship tasks. ECFPs are often considered to be non-invertible due to the way they are computed. In this paper, we present a fast reverse-engineering method to deduce the molecular structure given revealed ECFPs. Our method includes the Neuraldecipher, a neural network model that predicts a compact vector representation of compounds, given ECFPs. We then utilize another pre-trained model to retrieve the molecular structure as SMILES representation. We demonstrate that our method is able to reconstruct molecular structures to some extent, and improves, when ECFPs with larger fingerprint sizes are revealed. For example, given ECFP count vectors of length 4096, we are able to correctly deduce up to 69% of molecular structures on a validation set (112 K unique samples) with our method.

## Abstract 2

Protecting molecular structures from disclosure against external parties is of great relevance for industrial and private associations, such as pharmaceutical companies.

## Graphical abstract

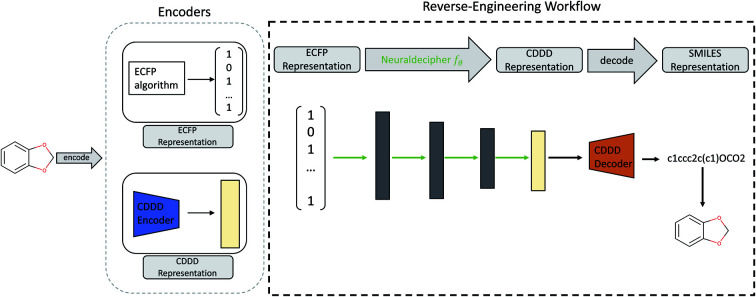

## 1 Introduction

The data protection and privacy of molecular structures are of crucial importance for industrial and private sectors, especially for the pharmaceutical industry. As the process of drug discovery is known to last at least a decade (10–20 years),^2,3^ pharmaceutical companies have utilized computational methods in the early stage to accelerate the generation of promising drug candidates that are active against a biological target, and the enrichment of chemical libraries for subsequent screening and analysis.

Molecular descriptors and fingerprints play a central role in computer-aided drug discovery, *i.e. in silico de novo* drug design, as they capture chemical information of the molecular structure as a vector of numbers that can be utilized for predictive modeling in several cheminformatic tasks.^4^ In quantitative structure–activity (QSAR) modeling, the aim is to model the relationship between compound and biological or physico-chemical endpoints. One biological endpoint is usually the binding affinity of a drug candidate against a protein target. Because drug candidates with high binding affinity can still fail in later phases of clinical trials due to poor pharmacokinetic and toxicological (ADMET) profiles, modeling ADMET endpoints such as solubility or melting point, is nowadays also considered in *in silico de novo* drug design at early stages.^5^

Securely exchanging chemical data without revealing the molecular structure is especially nowadays of great importance, as sharing data such as fingerprints and/or measured endpoints between research groups within academia or private sectors through collaborations is often accomplished to improve drug discovery.

An example for a large-scale collaboration is the MELLODDY (Machine Learning Ledger Orchestration for Drug Discovery) project,^6^ an Innovative Medicine Initiative (IMI) project by the European Union with a total funding of 18.4m EUR (2019–2022) including collaborations between pharmaceutical companies, research groups from universities but also small and medium-sized enterprises (SMEs).

Reconstructing the molecular structure that matches given chemical property values is a traditional (optimization) problem and often referred to as inverse-QSAR. One of the most commonly used molecular fingerprints in QSAR is the circular extended-connectivity fingerprint (ECFP).^7^ The ECFP has found many scientific applications starting from virtual screening and similarity search^8,9^ to biological target prediction,^10^ proteochemometric modeling^11^ and ADMET endpoint modeling.^12^

The topological ECFP representation is a refinement of the Morgan algorithm^13^ and usually hashed and folded into a fixed size 1024, 2048 or 4096 sparse bit or count vector to further utilize for predictive modeling tasks. During the fingerprint creation, the ECFP algorithm considers the atom environment, based on the maximum number of atomic neighbors, *i.e.* bond diameter *d*, and iteratively hashes the concatenated (unique) features to a new integer feature. Since the hash function is mapping randomly and uniformly to a 2^32^-size space of integers, the ECFPs are often considered to be non-invertible.^14^

In a study published by Kogej *et al.*^15^ in 2013 between the two large pharmaceutical companies AstraZeneca and Bayer AG, the extended-connectivity fingerprints (ECFP4) were exchanged among the two companies to subsequently do a nearest-neighbor search to enrich chemical libraries by the exploration of chemical space for prospective high-throughput-screening experiments. The choice for using the 2D binary molecular fingerprint was mainly referenced to the loss of intellectual property and competition issues in case a direct comparison of the two large collections of 1.41 M (AstraZeneca) and 2.75 M (Bayer AG) compounds was opted. It was thought that the ECFP4 ‘kept the molecular structures of both parties confidential, and in combination with a joint assessment workshop [they] could mitigate any concerns around intellectual property, reverse engineering or structure disclosure that would restrict individual scientists in project work’.^15^

The Joint European Compound Library (JECL)^16^ between 2013 and 2018 is another IMI collaboration accomplishment of seven pharmaceutical companies as well as academic research groups and SMEs to accelerate drug discovery on a precompetitive stage which resulted in a compound library of approximately 500 K small molecules for further screening. Among the 500 K compounds, 312 K non-commercial unique samples originate from pharmaceutical companies which were converted to ECFP6 and shared among the contributing pharmaceuticals as analyzed by Besnard *et al.*^17^ Similar to Kogej *et al.*, the ECFP6 was chosen by means of structure-free comparison without disclosure of proprietary information.

The initial combined library of pharmaceutical companies was further utilized for focused library design by academic institutions and SMEs to add *in silico* generated compounds to increase and reach the final library size of 500 K compounds.^18^

In this paper, we describe a method to reverse-engineer the extended-connectivity fingerprint and deduce the molecular structure of the compound. A simple approach to counteract the reverse-engineering could be obtained by permuting all the indices of the ECFP representation of a dataset with an arbitrary indexing, on which any predictive model or analysis on that dataset can still be trained and achieved. However, when working in a collaboration, such as the MELLODDY project, or the previous study by Kogej *et al.* between AstraZeneca and Bayer AG or the JECL, combining several databases of (permuted) shared fingerprint descriptors to do successive analyses inevitably requires to share the permutation matrix as well. By sharing the reindexing scheme, we return to our initial position of our motivation on the reverse-engineering of compounds based on ECFPs.

Related work analyzes to what extent chemical descriptors can be shared until molecular structures can be reverse-engineered. Those studies focused on the disclosure of physico-chemical properties and topological indices. In Masek *et al.*,^19^ the authors use an iterative genetic algorithm (GA) to suggest molecular structures that have the same chemical descriptor value(s) as a target compound. The genetic algorithm proceeds with suggested structures that match the descriptor value(s), *i.e.* minimize a total fitness function, which takes several descriptor values into account. The authors, however, test their method only on 100 selected target compounds and merely consider descriptors describing molecules that adhere to the Lipinski rule of five, or combination of BCUT descriptors and the MACCSkey fingerprint.^20,21^ Using their genetic algorithm, they obtained a high number of false positives – molecular structures that match the descriptor values but are in fact not the real molecular structure. A similar approach to Masek *et al.*, but not in the context of deducing molecular structures, is done by Winter *et al.*^22^ In their work for optimizing compounds in a drug discovery endeavor, the authors combine *in silico* prediction of molecular properties with an *in silico* optimization algorithm to suggest molecules that satisfy, or even positively improve, the desired characteristics defined by the user.

Faulon *et al.*^23^ proposes a stochastic and deterministic reverse-engineering algorithm to deduce the molecular structure from simple topological indices such as shape^24^ and connectivity^25^ indices, the Wiener^26^ and Balaban *J* and *J*_t_ distance indices^27^ as well as their developed atomic signature descriptor.^28^ In their analyses, the authors define the degeneracy as the number of structures having the same descriptor value in a given chemical database. From a computational point of view, descriptors with a high degeneracy are assumed to be safe to exchange, as those descriptors correspond to an 1-to-N mapping. This intuition becomes clear when the molecular weight (MW) is exchanged. Given the molecular weight, many possible molecular structures can be deduced. Similar to the work of Masek *et al.*, combining more chemical descriptors can improve the success rate of deciphering the true molecular structure. In their studies however, only 1000 compounds out of PubChem^29^ were randomly selected for reverse-engineering and their best method achieves a reconstruction accuracy of 12.2% with drawbacks in computation time on (local) CPUs.

Recent work from Kotsias *et al.*^30^ and Maragakis *et al.*^31^ in conditional *de novo* drug design utilize the ECFP representation of compounds as input (seed) with additional bioactivity labels to narrow and navigate the generative process towards chemical regions of interest. They train a generative model to sample novel compounds that satisfy the bioactivity condition and are to some degree similar to the input ECFP seed. Their study reveals that the trained generative models are able to sample compounds that correspond to the input seed.

The motivation of our work differs from Kotsias *et al.* and Maragakis *et al.*, as we want to train a model that learns the relationship between ECFP and its corresponding molecular structure, in contrast to the aforementioned work, that aims to generate new compounds, and by chance can reconstruct the compound that corresponds to the input ECFP.

One common evaluation approach for *de novo* molecular design methods is the rediscovery task of selected compounds based on their extended-connectivity fingerprints. The rediscovery task is methodologically different from our approach, as the rediscovery task aims to evaluate whether a generative model, trained on a given dataset, can sample selected (target) compounds that have been intentionally excluded from the training set. By successfully achieving the rediscovery of target compounds by the generative model, its goodness among the ability to sample accessible real-world compounds is strengthened. The GuacaMol benchmark by Brown *et al.* implements the rediscovery task as one benchmark among many goal-directed benchmarks, to assess the quality of SMILES-based generative models to retrieve the three target compounds Celecoxib, Troglitazone and Thiothixene.

**Fig. 1:**
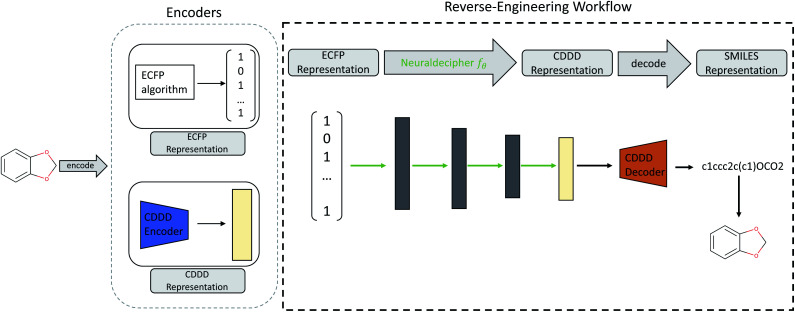
Illustration of the reverse-engineering workflow. Given an ECFP representation (here exemplary as bit-vector), we predict the corresponding cddd-representation and utilize the fixed decoder network from Winter *et al.* to obtain the SMILES representation. Therefore the Neuraldecipher learns the mapping between the two encoded molecular representations. Trainable parameters for the Neuraldecipher are displayed as green arrows, while black arrows correspond to operations that are fixed and not optimized during training.

Our main contributions are two fold. First, we describe the Neuraldecipher (illustrated in Fig. 1), a fast method to decipher the circular extended-connectivity fingerprint (ECFP)^7^ to their molecular structure as SMILES representation^33^ by formulating the reverse-engineering task as a machine learning (ML) problem. Next, we show how our method is performing on several configurations for the ECFP, based on selected length *k* of the fingerprint and bond diameter *d*. These studies attempt to answer the question, to what extent ECFP can be securely shared, until our proposed method can fully reconstruct the molecular structure on unknown fingerprints. We want to emphasize the importance for the protection of intellectual property and raise awareness that exchanging possibly invertible fingerprints can cause damage on a competitive level for private institutions, such as pharmaceutical companies. Since it is nowadays common for private and public institutions to work in joint collaboration to accelerate drug discovery as seen in JECL or MELLODDY, the development of secure and appropriate molecular fingerprints for common downstream tasks in computational chemistry is desired. Our study shows how to reverse-engineer extended-connectivity fingerprints and should motivate research groups to start of a new field in cryptographic chemistry.

## 2 Methods

One computational way to achieve the reconstruction would be to compare the given ECFP sample against a large accessible chemical library, where the mapping from ECFP to SMILES representation is known. The molecular structure could then be deduced by either performing an identity check of a given ECFP and the corresponding chemical library, and then returning those samples which match the target ECFP. If the ECFP representation cannot be found in the chemical library, the ECFP should be screened against that chemical library by computing pairwise similarities between the target ECFP and each sample of the reference library. A similarity measure could be the Tanimoto similarity of the respective ECFP pair. Deducing the molecular structure is then achieved by returning those pairs with highest Tanimoto similarity *τ* satisfying a defined treshold, *e.g. τ* > 0.90.

We formulate the reverse-engineering task as a machine learning problem with the goal to predict the molecular structure given an observed ECFP sample. Our reverse-engineering method is a two-step approach and utilizes the continuous and data-driven molecular descriptor (cddd), a neural network model for the generation of lower-dimensional vector representation of molecular structures.^1^ This model utilizes a recurrent autoencoder trained on the task of translating SMILES representation of compounds into their canonical form. Translation works as follows: first, the encoder model translates the input SMILES representation into the cddd-representation, a 512-dimensional vector representation for compounds, that have been shown to be effective on QSAR prediction and virtual screening tasks.^1^ Second, the decoder network translates the cddd into the canonical SMILES representation. The SMILES notation is a representation that encodes the topological molecular graph into a linear string of symbols.

Our goal for reverse-engineering is to predict the corresponding cddd vector, given an input ECFP sample. Once we have predicted the cddd vector, we can deduce the molecular structure by utilizing the fixed decoder network, which returns the SMILES representation. Our proposed method has the advantage that we obtain a regression model that is able to predict the molecular structures of ECFP samples more efficiently in a one-shot scenario, as opposed to an autoregressive model that predicts the SMILES representation given an input ECFP. By utilizing the pretrained CDDD model, the Neuraldecipher does not have to learn its own representation of chemical structures and to reconstruct SMILES strings with the correct syntax as illustrated in Fig. 1.

To obtain the SMILES representation, the decoder recurrent neural network (RNN) from Winter *et al.* takes the predicted cddd vector as input and feeds it into a fully-connected layer whose output is split into three parts to initialize three stacked recurrent layers. The output of the decoder network's RNN is a sequence of probability distributions of the different possible characters over the defined SMILES token by Winter *et al.* The deterministic decoder RNN applies a left-to-right beam search^34^ with a beam-width of 10 to obtain the final SMILES representation.

### 2.1 Neuraldecipher model

The Neuraldecipher model is a standard feedforward neural network with fully connected layers. Let 
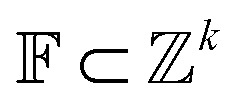
 be the ECFP-space with dimension *k*, where *k* is the length of the folded extended-connectivity fingerprint. Depending on bit or count extended-connectivity fingerprints, the entries of the ECFP are either populated with {0,1} or positive integers 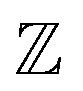. The CDDD-space 
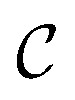
 is a bounded and compact 512-dimensional space, *i.e.*
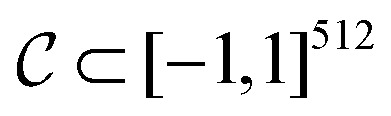
. The Neuraldecipher *f*_*θ*_ is a regression model, mapping from ECFP-space to the corresponding CDDD-space, *i.e.*
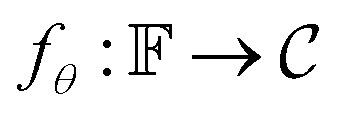
, where *θ* is the set of trainable model parameters. Fig. 1 illustrates the general reverse-engineering workflow.

The training of the Neuraldecipher is done *via* minimizing the distance *l*(*d*) = *l*(cddd_true_ − cddd_predicted_), where *l* is the logarithmic cosine–hyperbolic function, which is a similar loss function as the *L*_2_ squared-error loss. The logarithmic cosine–hyperbolic function is defined as1
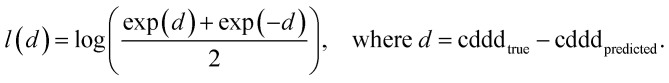


The number of hidden layers and corresponding hidden neuron units depend on the length of the input ECFP, *i.e. k* and will be discussed in the results Section 3.

We used ADAM optimizer with initial learning rate of 10^−4^ and 5 × 10^−4^ as weight decay coefficient. We trained the Neuraldecipher model for 300 epochs with a batch-size of 256. The learning rate was updated and multiplied by 0.7 according to a plateau scheduler with a patience of 10 epochs with respect to the validation metric. Additionally, we applied early stopping with a patience of 50 epochs with respect to the validation metric. Throughout all training experiments, the validation metric was the loss on a validation set.

### 2.2 Datasets

The data used in this study were extracted from the ChEMBL25 database^35^ and consists of 1, 870, 461 molecular structures. We used RDKit^36^ to retrieve the canonical SMILES representation and removed stereochemistry. We also removed duplicates and filtered with RDKit using the same criteria as done by Winter *et al.*: only organic molecules, molecular weight between 12 and 600 Da, more than 3 heavy atoms and a partition coefficient log *P* between – 7 and 5. Furthermore, we stripped the salts and only kept the largest fragments. After this procedure, our processed dataset contains 1, 526, 990 unique canonical SMILES representation. Yet, across many applications, machine learning models often fail to generalize when tested on data distributions different from training data.^37^ In order to check whether our model is not overfitting and motivate a real-world scenario, we clustered the processed SMILES dataset into 10 groups. The clusters were obtained by first computing the MACCSkey fingerprint^21^ for each SMILES representation using RDKit, and then utilizing sklearn's KMeans clustering implementation^38^ on the MACCSkey fingerprints. To obtain training and validation set, we computed the average pairwise distances between each of the 10 cluster centroids. The validation cluster was then selected by retrieving the cluster (in our case, cluster 7) whose centroid was on average the most distant to the other cluster centroids. Finally, our training set consist of 1, 414, 658 samples and validation set of 112, 332 samples. We call this splitting procedure cluster split. To evaluate how our model performs on a random split, we randomly divided the processed dataset into training and validation set with the same validation set size as in the cluster split scenario. Training of the model is done with the training set and model selection is based on the evaluation on the validation set.

**Fig. 2:**
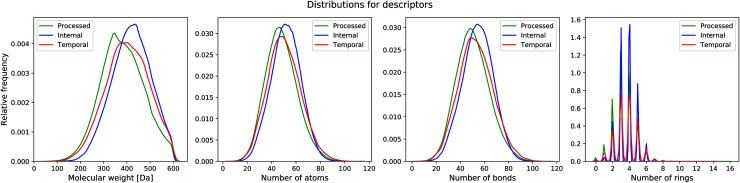
Distribution of molecular properties (molecular weight, number of atoms, number of bonds, number of aromatic rings) in the different datasets.

We also test our model on two unseen sets that have no overlap with the training set. The first set is the filtered ChEMBL26 temporal split (with 55, 701 unique compounds) and the second set consists of compounds from one of our internal databases (with 478, 536 unique compounds). The ChEMBL26 temporal split contains compounds that are novel in the ChEMBL26 database,^39^ when compared to ChEMBL25. For the internal set, we randomly sampled 500, 000 compounds from one of our processed databases that have no overlap with the ChEMBL25 set. We applied the same preprocessing filter as done before for both datasets. Dataset statistics for the processed, internal and temporal sets are listed in Table 1 and distribution plots displayed in Fig. 2.

**Table 1:** Dataset statistics for the processed, internal and temporal datasets. The values listed are the mean (standard deviation) values for each descriptor. The descriptor values were computed with RDKit. The last column displays the number of unique samples in each dataset

Dataset	Mol. weight	Num. atoms	Num. bonds	Num. rings	Number of samples
Processed (train/valid.)	380.70 (90.76)	48.18 (12.98)	50.53 (12.64)	3.37 (1.24)	1, 414, 658/112, 332
Internal	418.85 (82.89)	51.73 (12.04)	54.48 (12.62)	3.76 (1.06)	478, 536
Temporal	401.75 (91.53)	50.38 (12.54)	53.03 (14.21)	3.66 (1.29)	55, 701

#### 2.2.1 ECFP data

To analyze to which extend folded ECFPs can be securely exchanged, we created ECFP bit and count vectors for the lengths *k* ∈ {1024, 2048, 4096, 8192, 16 384, 32 768}. The bond diameter *d* was selected as *d* = 6, leading to ECFP_6,*k*_ bit- and count fingerprints. Since the collision of bits/counts with increasing fingerprint size decreases, more information about the molecular structure is preserved in the ECFP. Following this thought, our hypothesis is that deciphering molecular structures on larger ECFP size becomes more accurate, as the folded ECFP adheres a smaller information loss. To gain insight on how the model is performing on fingerprints created with different bond diameters and folded onto a fixed length, we calculated ECFPs of length 4096 and bond diameters {4, 8, 10}.

#### 2.2.2 CDDD data

To train and validate our method, we obtained the cddd vector representation by utilizing the encoder network of Winter *et al.* for each unique SMILES representation in our processed datasets, *i.e.* training and validation set (Table 1).

## 3 Results

For each ECFP setting introduced earlier, we conducted a hyperparameter search by defining possible parameters and searched for the optimal parameters using grid- and random search with a maximal number of 200 trials. We refer to the ESI† for description of the hyperparameter optimization and report the general model architecture and training procedure in the following. Each hidden layer consists of three consecutive operations: affine linear transformation, batch-normalization, and ReLU activation. We tested other activation functions like leaky ReLU, ELU and SoftPlus in the initial experiments, but found ReLU to be superior to the aforementioned non-linearities.

We applied at least 3 hidden layers and decreased the hidden neuron units to 512, followed by the output layer with 512 neurons and applied tanh non-linearity as output activation, since the cddd-vectors are bounded within [−1, 1]. All models were implemented in PyTorch.^40^

### 3.1 Degeneracy analysis

**Fig. 3:**
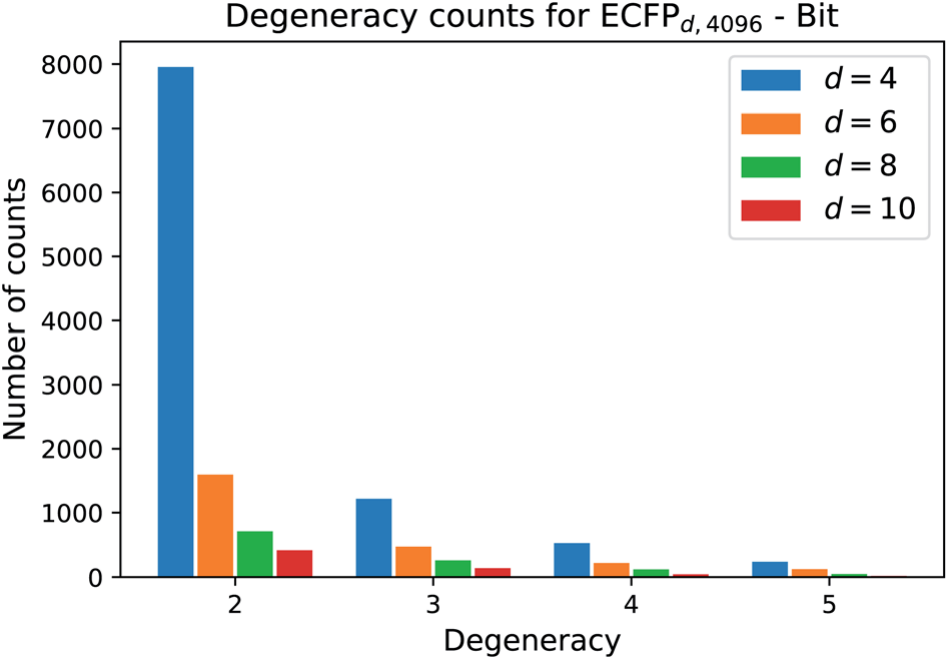
Frequency count for each degeneracy. As the bond diameter *d* increases, the count for each degeneracy decreases, *i.e.* there are more unique ECFP samples. The barplot displays the counts for the degeneracies [2, 3, 4, 5]. Degeneracies larger than 6 are not displayed, since the frequency that 6 different structures map to the same ECFP is small.

One natural question that arises with any molecular descriptor or fingerprint is the degeneracy. Recall that molecular weight as descriptor has a high degeneracy, since many compounds can correspond to a certain molecular weight. As the ECFP algorithm iteratively maps atomic environments to features, we believe that the computed ECFP sets from our processed dataset (1.4 M compounds) contains many unique samples with increasing bond-diameter *d*. Generally speaking, the larger the bond-diameter *d* is selected, the more local features of a compound are used to create the final fingerprint. To analyze the uniqueness of ECFPs, we computed the degeneracy for each ECFP dataset obtained with increasing bond-diameter *d* and show the analysis for the bit ECFPs with length 4096 in Fig. 3.

The horizontal axis states the degeneracy, *i.e.* it flags the presence of duplicate, triplicate, and so on. Since we want to count the number how often duplicates, triplicates, and so on, occure, we excluded the degeneracy of 1, *i.e.* the number of unique ECFP samples that occure only one time in the processed dataset.

A degeneracy of 3 means that 3 different structures have the same ECFP. Since this can happen multiple times, the vertical axis counts the occurence of each degeneracy within the processed dataset. As the bond diameter *d* increases for the ECFP_*d*,4096_ bit-vectors, the number of unique samples increases, *i.e.* the degeneracy counts for duplicates, triplicates, *etc.* decrease. A higher bond diameter in the ECFP algorithm leads to more uniqueness as more structural information is captured when iterating over larger atom environments (see Table 2).

**Table 2:** Illustration of non-unique samples for the ECFPs created with length 4096 and increasing bond diameter *d*. The first column describes the ECFP setting with bond diameter *d* and length *k*. The second column states the number of non-unique samples for ECFP-bit vectors, whereas the third column reveals the number of non-unique samples for ECFP-count vectors. To illustrate that the number of non-unique ECFPs is mainly influenced by the bond diameter *d* (for variable length *k*), the results for ECFP_6,1024_ with length 1024 and bond diameter 6 is also listed

ECFP	# non-unique bit	# non-unique count
ECFP_4,4096_	14, 382	2, 671
ECFP_6,1024_	4, 569	232
ECFP_6,4096_	4, 481	232
ECFP_8,4096_	2, 509	14
ECFP_10,4096_	1, 005	6

The number of non-unique samples for a fixed diameter *d* = 6 and increasing vector length *k* does not differ much, as the ECFPs (folded into fixed-length vectors of size *k*) represent the same structure in a larger fingerprint vector.

We refer to the ESI† for a detailed list of non-unique samples in each setting.

Since the encoded cddd-representation benefits from an injective mapping given SMILES in contrast to the ECFPs, an interesting bound to analyze is the distance between encoded cddd-representations, where the mapping from ECFP to SMILES is non-unique (we call that set of SMILES tuples *S*_*d*_). Generally, the impact of the non-uniqueness from ECFPs can compromise the training of the Neuraldecipher in two scenarios. In the first preferable scenario, the (average) distance between cddd's encoded from *S*_*d*_ tuples is low. That means low distortion in the corresponding CDDD-space when learning the mapping from ECFP-space to CDDD-space. The second scenario includes a larger average distortion and could degrade the training of the Neuraldecipher, since learning the mapping from ECFP-space to CDDD-space is perturbed as the model encounters ECFP samples that map to diverse cddd-representations. To analyze the two possible scenarios, we retrieved the set of SMILES *S*_*d*_ that includes tuples (*i.e.* duplicates, triplicates, *etc.* see Fig. 3), of SMILES representation that map to the same ECFP. We retrieved the corresponding cddd's for each tuple set of *S*_*d*_, and calculated the average cosine distance of each pair in the tuple sets.

**Fig. 4:**
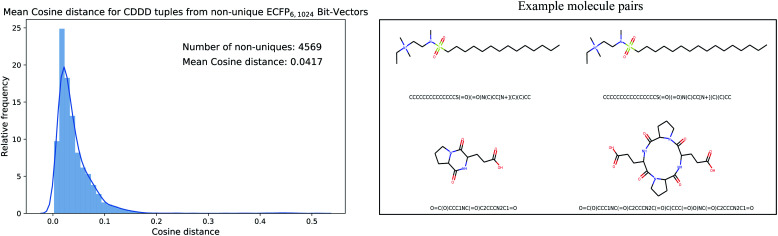
Mean cosine distance between cddd-representations that belong to the same set of SMILES that map to the same ECFP representation. On average, the cosine distance is small with 0.0417.

Fig. 4 illustrates the results for the ECFP_6,1024_ bit-vector setting. The ambiguity of binary ECFPs with different SMILES representation does not cause a large distortion in the corresponding CDDD-space, as the unsupervised learned representation maps structurally very similar SMILES into close space as indicated in the average cosine distance of 0.0417. The right plot in Fig. 4 illustrates two randomly selected pair of molecules for the first scenario (low distortion, *i.e.* cosine distance ≤0.05) and second scenario (high distortion, *i.e.* cosine distance ≥ 0.20). However in the second scenario, the binary ECFP can misleadingly map to a representation, where the molecules are more different (the molecule pair in the second row of Fig. 4 has a cosine distance of 0.3335). Since the binary-ECFP only captures presence of certain atomic environments (and not counts, as opposed to count-ECFP) the molecules in the second row of the right plot in Fig. 4 correspond to the same ECFPs but refer to different cddd-representations with larger distortion.

### 3.2 Results and discussion

We trained separate Neuraldecipher models on a cluster and random split, for each ECFP setting. The ECFP setting was determined by bond diameter *d*, fingerprint length *k* and exposure of bit- or count ECFPs.

After training, for the final evaluation on the validation, internal and temporal dataset, we predicted the corresponding cddd-vectors and retrieved the SMILES representation by utilizing the decoder network from Winter *et al.*

### 3.3 Varying the length *k* for fixed diameter *d* = 6

The results for the ECFP_6,*k*_ bit-vectors with increasing length *k*, trained on cluster and random split are listed in Table 3.

**Table 3:** Results for reverse-engineering molecular structures based on ECFP_6_-bit vectors. To compute the average Tanimoto similarity for all lengths, we first calculated the ECFP_6,1024_ bit-vectors for the true and reconstructed SMILES and then parsed the tuple into RDKit's Tanimoto similarity implementation. We selected a fixed ECFP configuration across all lengths *k*, to have a proper and comparable evaluation on the validation (Valid.), internal (Inter.) and temporal (Temp.) datasets. Larger values up to 100 are better

*k*	Cluster split						Random split					
	Reconstruction [%]			Tanimoto [%]			Reconstruction [%]			Tanimoto [%]		
	Valid.	Inter.	Temp.	Valid.	Inter.	Temp.	Valid.	Inter.	Temp.	Valid.	Inter.	Temp.
1, 024	12.14	11.32	13.34	47.08	45.31	46.84	28.70	12.11	14.14	60.64	40.30	47.60
2, 048	18.85	15.85	18.04	53.65	49.68	51.17	37.87	16.34	18.81	67.11	50.26	51.87
4, 096	32.90	25.08	28.12	63.02	57.06	59.11	57.35	25.30	28.43	79.36	57.39	59.55
8, 192	48.83	37.14	39.98	74.25	66.45	68.24	72.91	36.84	39.81	88.01	66.57	68.33
16, 384	57.85	44.64	47.38	79.80	71.86	73.46	79.79	46.22	48.86	91.30	72.96	74.34
32, 768	59.04	45.81	48.31	80.77	72.84	74.21	80.02	46.92	49.66	91.40	73.35	74.76

The reconstruction columns in Table 3 correspond to the accuracy of binary string matching between true input SMILES representations and deduced SMILES representations. Hence, the reconstruction refers to the accuracy of correctly deducing the exact molecular structure given the ECFP_6_-bit vectors. The Tanimoto columns state the average Tanimoto similarity between true input SMILES and deduced SMILES representations. To compute the Tanimoto similarity, we retrieved the ECFP_6,1024_ bit fingerprints of true and deduced SMILES and utilized RDKit's Tanimoto similarity implementation. We included the Tanimoto similarity as proxy for the goodness of reverse-engineering, since our model might fail to fully deduce the exact molecular structure, but is still able to reconstruct (structurally) similar compounds that resemble the true compound, which could be optimized in a subsequent task.

Considering that we are using the decoder network to retrieve the reconstructed SMILES representations of predicted cddd-representations, the validity of reconstructed SMILES, *i.e.* if the string representation follows the SMILES grammar, is of great importance, especially in generative modeling.^32^

In all experiments, the SMILES validity on the test datasets (validation, internal, temporal), was most of the time around 98%. We refer to the ESI† for a detailed view of the validity for each configuration. All metrics were computed using the validation (Val.), temporal (Temp.) and internal (Inter.) datasets, which the models have not seen during training.

As expected, models trained on the random split perform better than models trained on the cluster split, when deducing molecular structures from the validation dataset. For example, the model for the ECFP_6,1024_ is able to correctly deduce 12.14% from the validation dataset when trained on the cluster split. The reconstruction for the cluster split is smaller because the validation dataset contains compounds which likely lie in a chemical space, the model has not seen before during training. When the model is trained on a random split, 28.70% of the validation dataset can be correctly reverse-engineered. For the internal and temporal datasets, the performance for cluster split and random split are almost similar along all models. This insight is normal and expected, as the data distributions from the internal and temporal sets generally differ from the processed ChEMBL25 dataset.

One of our hypotheses was that the probability of reverse-engineering molecular structures from folded ECFPs increases with larger size, as the ECFPs are less prone to information loss due to hash collision. This is confirmed by our experiments (Table 3), as models trained with larger ECFP_6_ input bit-vectors are more capable to correctly deduce the molecular structure in all evaluation datasets. Increasing the ECFP size from 16, 384 to 32, 768 does not improve the performance very much, as the information loss through the hash collision is small. For an analysis on the hash collision for the analyzed fingerprint lengths, we refer to ESI.†

Our reverse-engineering workflow has the benefit of fast computation for the intermediate cddd-representation. The elapsed time for one forward pass of 1 M compounds, when predicting the cddd-representation given varying ECFP-representations, amounts to approximately 5 seconds given ECFPs of length 1024, and up to 100 seconds for ECFPs of size 32, 768. Using the cddd-decoder RNN model to obtain the SMILES representation requires more time due to the nature of sequential models and integration of beam-search. Decoding 1 M cddd-representations back to SMILES representations requires around 38 minutes. The complete reverse-engineering workflow of 1 M compounds takes about 39 minutes and 40 seconds in case the ECFP-representation of length 32, 768 is used as input for the Neuraldecipher. All computations were performed on a single modern Nvidia Tesla V100 GPU.‡‡This computation would cost around 
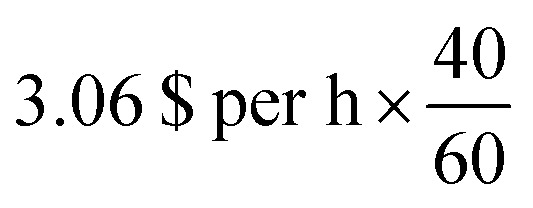
, h = 2.04 per $ on a p3.2× large AWS instance on demand.

**Fig. 5:**
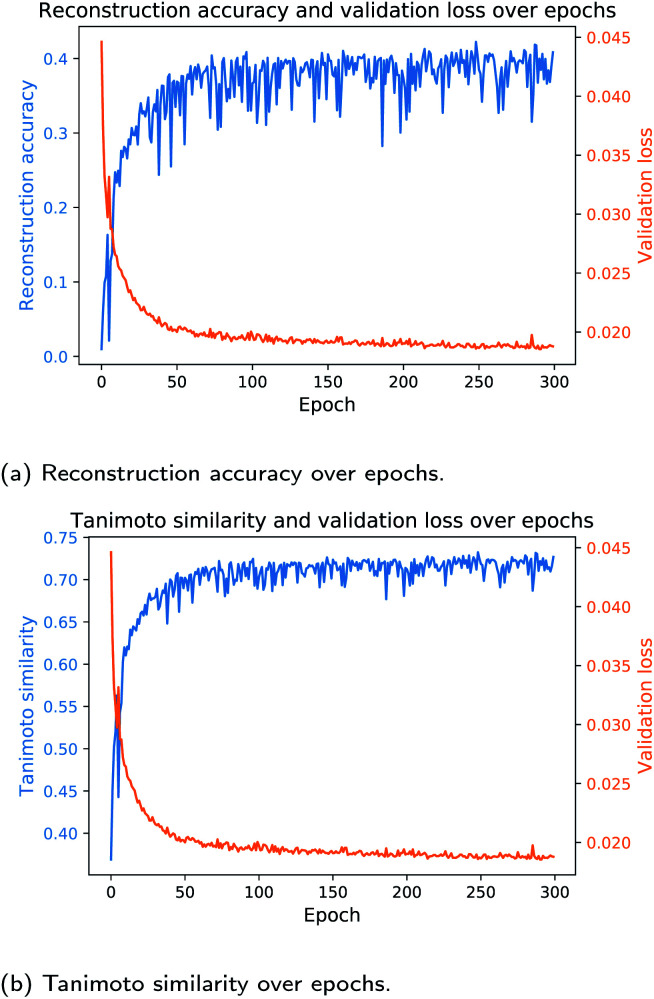
Progress of the ECFP_6,4096_-count model during training for the reconstruction accuracy and Tanimoto similarity over epoch. Each plot shows the corresponding metric and the validation loss (cluster split validation) after each training epoch.

In the next study, we trained the models from Table 3 with the same network architectures for each fingerprint length on the ECFP_6_-count vectors. As the ECFP_6_-count vector preserves more information about a molecular structure than the corresponding ECFP_6_-bit vector, the models trained on the ECFP_6_-count vectors are expected to perform better than models trained on bit vectors only. Table 4 shows the results of this study. Training the Neuraldecipher models on ECFP_6_-count vectors yields better performance metrics as seen in Table 4 compared to Table 3. For the model trained on 1024 length ECFP, the correct reconstruction of molecular structures in the validation dataset improves to 22.49% for the cluster split model when trained on count vectors as opposed to 12.14% when trained on bit vectors. The conclusions made earlier for better performance with increasing fingerprint size are also reflected in the results in Table 4. With our reverse-engineering method, we are able to correctly deduce around 150 K compounds from the Bayer internal dataset (478 K samples) with 31.73% accuracy, when ECFP-count vectors of length 4096 are shared (see Table 4, random split). Considering that we only used publicly available data from ChEMBL to train the Neuraldecipher model, extra caution has to be paid when exchanging ECFPs with legitimate partners, as the protection of molecular structures is of importance for pharmaceutical companies. The validity of SMILES for all models is as before on average 98%. Since the learning rate scheduler and early stopping mechanism for model selection during training is only affected by the validation loss per epoch, we only computed the evaluation metrics in Tables 3 and 4 based on the final selected model. To observe the progress of evaluation metrics (*i.e.* reconstruction accuracy and Tanimoto similarity), we trained the Neuraldecipher on ECFP_6,4096_-count vectors on the cluster split for 300 epochs without early stopping and computed the corresponding metrics after each training epoch. Fig. 5 shows the progress of the reconstruction accuracy and Tanimoto similarity over epochs compared with the validation loss.

**Table 4:** Results for reverse-engineering molecular structures based on ECFP_6_-count vectors. To compute the average Tanimoto similarity for all lengths, we first calculated the ECFP_6,1024_ count-vectors for the true and reconstructed SMILES and then parsed the tuple into RDKit's Tanimoto similarity implementation. We selected a fixed ECFP configuration across all lengths *k*, to have a proper and comparable evaluation on the validation (Valid.), internal (Inter.) and temporal (Temp.) datasets. Larger values up to 100 are better

*k*	Cluster split						Random split					
	Reconstruction [%]			Tanimoto [%]			Reconstruction [%]			Tanimoto [%]		
	Valid.	Inter.	Temp.	Valid.	Inter.	Temp.	Valid.	Inter.	Temp.	Valid.	Inter.	Temp.
1, 024	22.27	16.92	19.41	61.39	57.59	59.06	38.29	17.85	20.90	71.11	58.13	59.42
2, 048	30.45	22.35	25.94	66.25	61.32	62.90	47.73	22.22	25.77	76.36	61.34	62.99
4, 096	41.02	29.98	34.61	72.58	66.43	68.52	66.61	31.73	36.22	85.98	67.61	69.59
8, 192	55.01	39.63	44.56	80.49	72.77	74.85	77.07	40.89	44.97	90.98	73.60	75.29
16, 384	62.42	46.47	50.61	84.30	76.83	78.44	80.02	46.02	49.48	92.45	76.69	78.05
32, 768	64.03	48.52	52.32	85.07	78.01	79.30	83.52	50.35	54.25	93.85	79.09	80.44

Fig. 5 shows that with decreasing validation loss, the reconstruction accuracy and mean Tanimoto similarity on the validation dataset increase. However, the reconstruction accuracy on the validation data (112, 332 samples) seems volatile and reaches on average 41%. Although the model is not capable to fully deduce the molecular structure, it is able to reconstruct on average compounds that have mean Tanimoto similarity of 72%.

A positive relationship between (1 – Tanimoto similarity) and validation loss in Fig. 5b is also shown in the analysis when plotting the Euclidean distance in the corresponding CDDD-space for true cddd and predicted cddd and plot it against (1 – Tanimoto similarity). We refer to ESI for more details.†

### 3.4 Varying the bond diameter *d* for fixed length *k* = 4096

The results in Tables 3 and 4 show that the performance on successfully reconstructing molecular structures improves, when the fingerprint length *k* increases and count-vectors instead of bit-vectors are shared. Our next study analyzes how our model performs on a fixed ECFP input length *k* = 4096 and varying bond diameter *d*.

As the bond diameter *d* in the ECFP algorithm determines the number of iterations per atom to capture structural information of atom environments, an ECFP generated with bond diameter *d*′ > *d* is a superset of the ECFP, that was created with bond diameter *d*. At each diameter, the fingerprint is the combination of features from the previous diameter, plus any new features discovered by that step.^7^ In other words, ECFP bit- or count vectors with a higher bond diameter *d*′ can capture more information and the entries of the fingerprint can be more populated with 1's or integers for bit- or count vectors, as opposed to ECFPs created by smaller bond diameter *d*. We selected the same network architecture from the ECFP_6,4096_ model and trained the model on ECFP_*d*,4096_ bit- and count vectors, where *d* ∈ {4, 8, 10}. The results for the experiments trained on cluster split and random split are listed in Table 5.

**Table 5:** Results for reverse-engineering molecular structures for ECFPs with fixed length of 4096 and increasing bond diameter *d* on the cluster- (cs) and random (rs) split. The results for ECFP_6,4096_ from Tables 3 and 4 are listed for completeness. To compute the Tanimoto similarity, we always computed the ECFP_6,1024_ count/bit-vectors for true SMILES and reconstructed SMILES representation in order to have a proper and comparable evaluation for all bond diameters. The first column states the ECFP with the bond diameter *d* and the flag for cluster (cs) or random (rs) split. Higher values up to 100 are better

ECFP	ECFP-count						ECFP-bit					
	Reconstruction [%]			Tanimoto [%]			Reconstruction [%]			Tanimoto [%]		
	Valid.	Inter.	Temp.	Valid.	Inter.	Temp.	Valid.	Inter.	Temp.	Valid.	Inter.	Temp.
ECFP_4,cs_	43.60	33.19	37.44	74.27	68.93	70.77	34.62	27.21	29.70	65.72	59.72	61.53
ECFP_4,rs_	68.92	34.23	38.78	87.40	69.76	71.60	60.98	28.22	30.98	82.01	60.32	62.20
ECFP_6,cs_	41.02	29.98	34.61	72.58	66.43	68.52	32.90	25.08	28.12	63.02	57.06	59.11
ECFP_6,rs_	66.61	31.73	36.22	85.98	67.61	69.59	57.35	25.30	28.43	79.36	57.39	59.55
ECFP_8,cs_	36.56	26.72	30.56	70.10	64.20	66.34	27.27	21.91	25.14	59.90	54.53	56.75
ECFP_8,rs_	60.21	27.09	31.17	83.09	64.59	66.50	53.03	22.22	25.52	76.70	54.83	57.15
ECFP_10,cs_	34.37	25.52	29.51	68.88	63.37	65.27	23.95	19.82	22.92	57.73	52.89	55.15
ECFP_10,rs_	59.52	26.56	30.98	82.58	64.18	66.33	51.52	21.42	24.55	75.41	53.96	55.94

The results in Table 5 go along with the finding that models trained on the random split (rs) perform better on the validation dataset, compared to models trained on the cluster split (cs). There seems to be no substantial difference between the performance on the internal and temporal datasets, when the model was trained on the cluster or random split. Models trained with count-vectors as input perform better than models trained with bit-vectors, as count-vectors preserve more information about the molecular structure.

However, we observe that the performance decreases with increasing bond diameter, regardless of which split the model was trained on. Recall that the unfolded ECFP with a larger bond diameter *d*′ is a superset of the unfolded ECFP with smaller bond diameter *d*, because more substructures are captured with higher bond diameter (*d*′ > *d*) during the fingerprint algorithm. So generally, the unfolded ECFP_*d*′_ captures more information than the unfolded ECFP_*d*_. Folding the ECFP_*d*′_ to a fixed length of 4096, *i.e.* to ECFP_*d*′,4096_, comprises a higher information loss due to hash collision. Note that we concluded a similar observation when studying the behavior for increasing fingerprint length *k*: with increasing fingerprint length *k*, less information was lost, and therefore the model performance increased (see Tables 3 and 4) for the ECFP_*d*,4096_. As a result of this, training the Neuraldecipher (with a fixed network architecture) on ECFP_4,4096_ representation as input, leads to better performance compared to the setting, when the input is ECFP_6,4096_. The performance decrease from diameter 8 to 10 is comparably small to the other differences (*i.e.* 4 to 6 and 6 to 8), as the unfolded ECFP_8_ representations are in most cases the same as the unfolded ECFP_10_ representations and folding these representations into fixed length of 4096 causes the same collision. For a detailed analysis on the hash collision, we refer to ESI.†

### 3.5 Comparison neuraldecipher against baseline

To further analyze the magnitude of Tanimoto similarity in the cluster validation dataset (112 K samples), we compare our method against a purely computation approach from virtual screening (referred as “Library-Analysis Baseline” and explained in the beginning of Section 2).

**Fig. 6:**
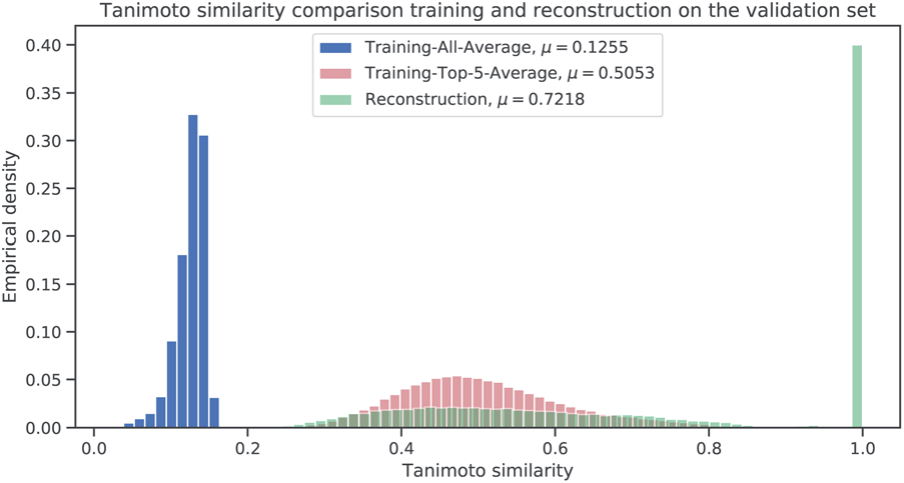
Histogram to illustrate the distributions for the Tanimoto similarity between true SMILES representations and retrieved SMILES representations from the average training (blue), baseline model (red) and our reconstruction (green) on the validation set (112 K samples).

For each validation sample, we calculated all pairwise Tanimoto similarities§§Based on ECFP_6,4096_ count-vectors. to each sample from the reference (library) training set (1.4 M samples). We then computed the average Tanimoto similarity for each validation samples by computing the mean of the aforementioned pairwise similarities (“All-Average”). For the baseline, we selected the top-5 references (training) samples with highest Tanimoto similarity (from the pairwise similarities) and computed the mean of the top-5 references for each validation sample (“Top-5-Average”). The “Top-5-Average” approach demonstrates a weak¶¶The baseline is weak because we are using the training set as reference library. baseline from compound-library analysis. The “All-Average” procedure aims to show, how similar a validation sample is on average to all samples from reference set, while the “Top-5-Average” procedure aims to show, how similar a validation sample is on average to the top-5 most similar samples from a reference set. Fig. 6 displays the Tanimoto similarity distributions between the “All-Average”, “Top-5-Average” and our Neuraldecipher model (trained on ECFP_6,4096_ count-vectors).

**Fig. 7:**
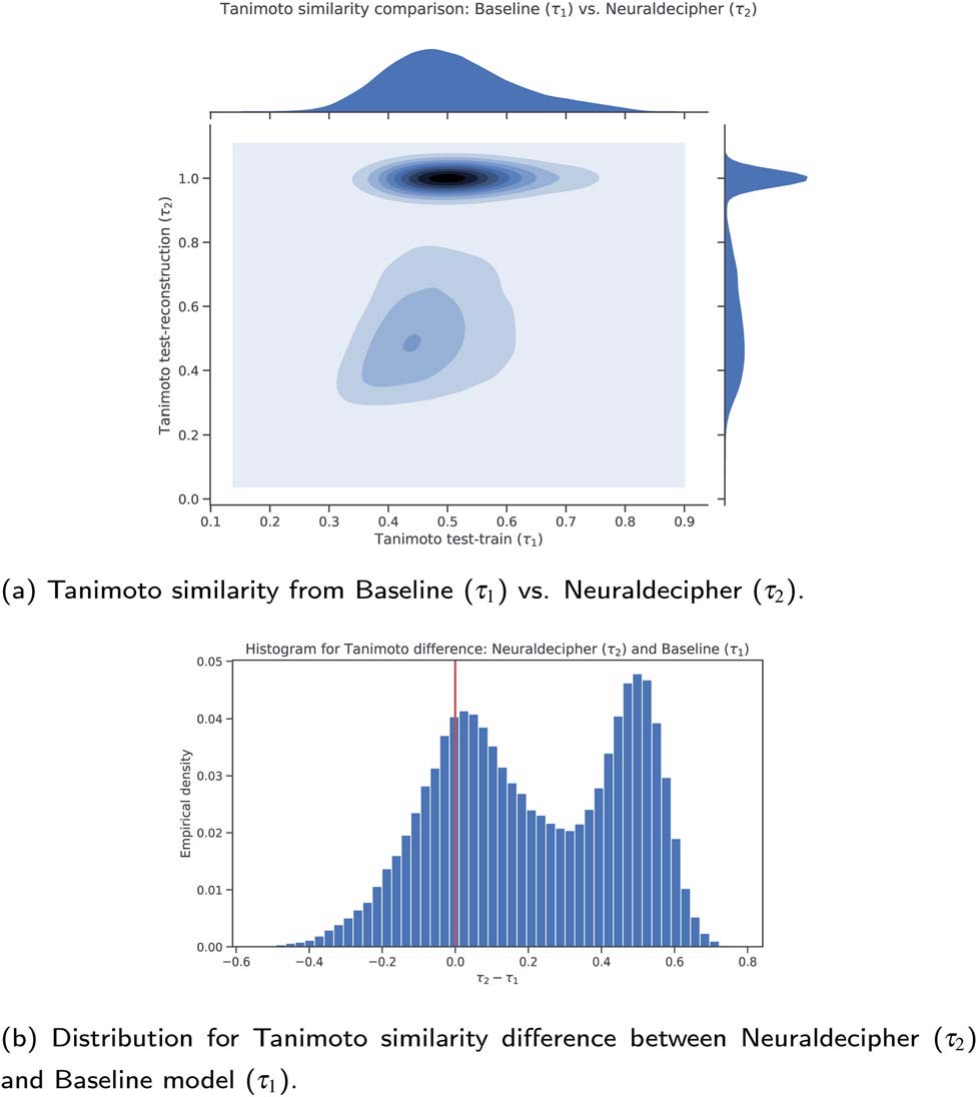
Comparison between the Neuraldecipher and baseline model wrt. the Tanimoto similarity on the validation dataset (112 K samples).

As expected and intended through the cluster split, the Tanimoto similarity between the validation and training (reference) set is small on average with 0.1255. The “Top-5-Average” baseline (shaded in red in Fig. 6) obtains a mean Tanimoto similarity of 0.5053 with fat tails approaching the Tanimoto similarity of 0.8. However, the baseline (and even Top-1-Average||||This would be the retrieved sample from the reference training set that is the most similar to a target sample.) cannot reconstruct the validation samples, *i.e.* reconstruction accuracy of 0. This means that the training (reference) set does not contain the “true” validation samples. This insight goes along with Table 2, displaying 232 non-unique samples for the ECFP_6,4096_ count-dataset. In that case, all non-unique samples are represented in the training (reference) set. Our Neuraldecipher however, achieves a reconstruction of 0.4102 and mean Tanimoto similarity of 0.7218. The fat tail of the Neuraldecipher Tanimoto similarity distribution along the horizontal axis between 0.4 and 0.7 (green curve in Fig. 6) is likely caused by the contribution of Top-5-Average samples. This means that our Neuraldecipher reconstructs structurally similar molecular compounds of that Tanimoto similarity range, because on average the best structures the model can learn from, also share this Tanimoto similarity of 0.5053. Therefore, there is less probability mass in the Tanimoto range of [0.8–0.9]. To compare the performance between the baseline and our method, we plotted the Top-5-Average Tanimoto similarities against the Tanimoto similarities of our reconstructions in Fig. 7.

Fig. 7a and b show that our proposed method performs on average better than the baseline method. Out of 112 K validation samples, our method can reconstruct 85 K samples that have a higher Tanimoto similarity than the baseline model, *i.e.* in 75.89% of all cases. This is illustrated in the contour plot in Fig. 7a and more clearly in the distribution plot in Fig. 7b for *τ*_2_ − *τ*_1_ > 0.0. To analyze the role of approximate reconstruction we retrieved the subset of samples where our reverse-engineering workflow returned compounds with Tanimoto similarity less than 1.0. We applied the non-parametric paired Wilcoxon rank-sum test with the null hypothesis that the sample distributions of Tanimoto similarities for our reverse-engineering workflow is equal to the baseline, and the alternative hypothesis that the sample distributions are not equal, *i.e. H*_0_: *τ*_2_ = *τ*_1_*vs. H*_1_: *τ*_2_ ≠ *τ*_1_. The Wilcoxon rank-sum test is highly statistically significant with a *p*-value of *p* = 1.1921 × 10^−7^ < *α* = 0.05, rejecting *H*_0_ at the 5 – % significance level and indicating that the sample distributions are not equal. The mean Tanimoto similarity of (0.5363 ± 0.1512) from our method suggests that it performs on average better than the baseline (0.4925 ± 0.1105) on the selected subset with around 66.7 K samples.

Furthermore, our reverse-engineering workflow benefits from faster computation. Recall that the baseline model requires the computation of *N* × *m* pairwise similarities, where *N* = 1, 414, 658 and *m* = 112, 332, which subsequently have to be sorted in decreasing order. The elapsed time for the baseline model approximately amounts to 3.75 hours using all cores of a 96-core CPU-machine. Our reverse-engineering workflow only requires approximately 5 minutes using one single Nvidia Tesla V100 GPU and achieves a better reconstruction accuracy.**The computation on 96-cores would cost around 4.128 $ per h × 3.75, h = 15.48 per $ on a m5a.24× large AWS instance on demand while our reverse-engineering workflow would only cost around 
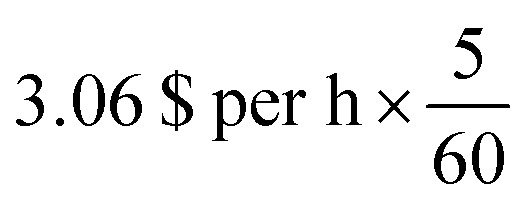
, h = 0.255 per $ on a p3.2× large AWS instance on demand.

One could argue to preserve a stronger baseline by increasing the size of reference library where the overlap between target set and reference library is potentially larger. However, computing pairwise similarities between target and reference library is computationally expensive and does not scale well. Additionally, one should consider that in real-life scenarios, the target dataset consists of in-house compounds from a private institution, that are of interest for reverse-engineering. In general, the baseline method is not able to infer the true compound, based on ECFP. However, if there is an overlap between target and reference library, this overlap is often caused by publicly available molecules, which are also present in open databases, as explored by Kogej *et al.* when screening the overlap between the AstraZeneca and Bayer AG libraries or other related work.^41,42^

## 4 Conclusion

In this work we proposed a reverse-engineering method to deduce the molecular structure given the extended-connectivity fingerprint (ECFP). To identify to what extend structures can be reconstructed, we tested our method on several fingerprint settings with varying length *k* and bond diameter *d* for the ECFP creation. In general, with increasing fingerprint size and count-vectors being revealed, our method is capable of better reconstructing molecular structures from large sets that our method has not seen before. We selected the ECFP to reverse-engineer from, as the ECFP is a commonly used fingerprint in QSAR and ADMET modeling and often considered as non-invertible. In case ECFP-count representations of length 4096 are exchanged (see Table 5), our method is able to correctly reconstruct up to 68.92% from a random subset of ChEMBL25 (112, 332 unique compounds), 38.78% from the ChEMBL26 temporal set (55, 701 unique compounds) and 34.23% from a random subset of one of our internal databases (478, 723 unique compounds). Although, and somehow fortunately, we did not reach a complete reconstruction on the test sets, due to information loss when folding the unfolded ECFP into fixed-length vectors, there might be small improvements by changing the training procedure. Since we have formulated the reverse-engineering task as a machine learning problem, and utilize neural networks as model class, finding the optimal network architecture and formulating different loss function for training entails the chance for better performance. We suggest that extended-connectivity fingerprints should be exchanged with precaution as this yields the potential to harm intellectual property and loss of competitive advantages since our method is capable to reconstruct molecular structures to some extent.

We hope we raised awareness about the danger when exchanging ECFP representations and motivated a new research field in cryptographic chemistry for the development of secure and appropriate fingerprints for cheminformatics.

## Availability

Source code of the proposed method is openly available at https://github.com/bayer-science-for-a-better-life/neuraldecipher.

## Conflicts of interest

There are no conflicts to declare.

## Supplementary Material

SC-011-D0SC03115A-s001
