# Supplementary material for: Neuraldecipher – reverse-engineering extended-connectivity fingerprints (ECFPs) to their molecular structures
Source: Chem Sci. 2020 Sep 11;11(38):10378–89. doi: 10.1039/d0sc03115a (PMC8162443; doi:10.1039/d0sc03115a)
Supplement: SC-011-D0SC03115A-s001 [file SC-011-D0SC03115A-s001.pdf]

Electronic Supplementary Material (ESI) for Chemical Science.
This journal is © The Royal Society of Chemistry 2020

Supplementary Information for Neuraldecipher - Reverse-engineering extended-connectivity fingerprints (ECFPs) to their molecular structures

Tuan Le,*,†,‡ Robin Winter,†,‡ Frank Noé,‡ and Djork-Arné Clevert*,†

†Department of Digital Technologies, Bayer AG, Berlin, Germany.

‡Department of Mathematics and Computer Science, Freie Universität Berlin, Berlin, Germany

E-mail: tuan.le2@bayer.com; djork-arne.clevert@bayer.com

Model architecture for varying fingerprint size

Table 1 displays the neural network architecture for each Neuraldecipher model. We applied at least 3 hidden layers and no dropout regularization throughout all of ours experiments. We tested dropout regularization with varying settings[1], but found that using dropout leads to inferior performance on the validation set, compared to models without dropout regularization. For hyperparamter tuning, we used the asynchronous Hyperband implementation of the open-source python library *tune*.[1] Figure 1 shows our selected logarithmic cosine-hyperbolid loss function (see Equation 1) and the standard L_2-loss.

[1]E.g., constant dropout probability of 0.1 for all hidden layers, applying dropout on further hidden layers as the input ECFP is sparse and dropping hidden units in the beginning might degrade the performance much, or exponentially decaying the dropout probability up 0.

Table 1: Architecture for each Neuraldecipher model. Each hidden layer consists of the composition of three operations, namely affine linear transformation, batch-normalization followed by ReLU activation. Each integer within the hidden layers bracket, indicates the number of hidden neurons in the hidden layer. The output layer consists of 512 neurons and is activated with Tanh. The last column (elapsed time) states the average duration of one forward pass of 1M compounds through the network for 10 forward passes.

ECFP input-size	Hidden layers	Output-size	Elapsed time [s]
1024	$[1024, 768, 512]$	512	5.46
2048	$[1024, 768, 768]$	512	7.39
4096	$[2048, 1024, 768, 512]$	512	10.61
8192	$[4096, 2048, 1024, 512]$	512	22.68
16384	$[8192, 4096, 2048, 1024]$	512	52.58
32768	$[8192, 4096, 2048, 1024]$	512	101.11

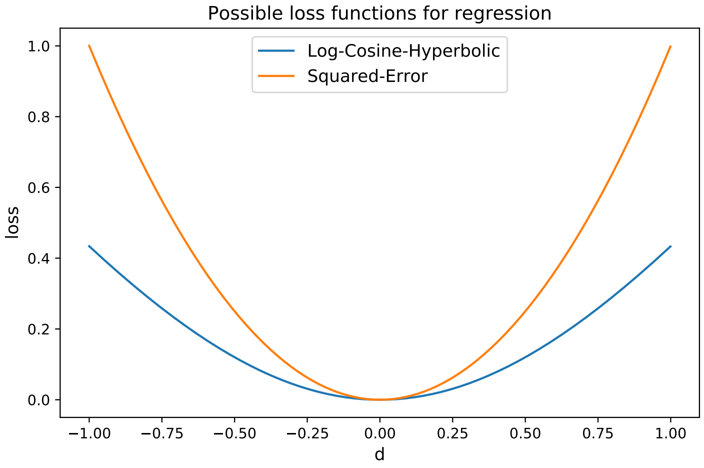

Figure 1: The logarithmic cosine-hyperbolic function and the classical L_2 loss function for regression. The first loss function penalizes stronger for $|d| \geq 0.25$.

$$l(d) = \log\left(\frac{\exp(d) + \exp(-d)}{2}\right), \text{ where } d = cddd_{\text{true}} - cddd_{\text{predicted}}. \tag{1}$$

Degeneracy analysis for $ECFP_6$ settings

The number of non-unique ECFPs for the processed dataset for training depends on the set bond diameter d. For the $ECFP_6$, i.e. generated with bond diameter $d = 6$ with increasing fingerprint length k, we computed the number of non-unique ECFP samples for the bit- and count vectors. The results are shown in Table 2. Given fixed bond diameter d, the number of non-unique samples does face large changes with increasing fingerprint length k. However, folding the ECFPs into smaller fingerprint sizes leads to more information loss, as explained in Section in detail.

Table 2: Number of non-unique samples within each ECFP6 dataset. As the bond diameter d is always the same with $d = 6$, the unfolded ECFPs are in all cases the same, and when folded into the fixed-vector length still remain "unique". The bond diameter is the decisive factor for a high number of degeneracy.

ECFP setting	# Non-unique Bit-ECFP	# Non-unique Count-ECFP
$ECFP_{6,1024}$	4569	232
$ECFP_{6,2048}$	4494	232
$ECFP_{6,4096}$	4481	232
$ECFP_{6,8192}$	4454	232
$ECFP_{6,16384}$	4454	232
$ECFP_{6,32768}$	4445	232

Validity on reconstructed SMILES in all experiments

Table 3: Validity [%] of reconstructed SMILES representation for the validation- (112, 322 unique samples), internal- (478, 536 unique samples) and temporal set (55, 701 unique samples) for the models trained on cluster and random split. In general, the validity of reconstructed SMILES is almost perfect with approximately 98 – 99%.

ECFP	ECFP-Count						ECFP-Bit					
	Cluster split			Random split			Cluster split			Random split		
	Valid	Inter	Temp	Valid	Inter	Temp	Valid	Inter	Temp	Valid	Inter	Temp
$ECFP_{6,1024}$	98.81	97.32	97.09	98.13	97.27	97.06	98.27	96.98	96.91	97.66	96.62	96.38
$ECFP_{6,2048}$	98.99	97.44	97.21	98.36	97.24	97.03	98.58	97.02	96.88	98.11	97.05	96.93
$ECFP_{4,4096}$	99.11	97.79	97.70	99.01	97.60	97.39	98.85	97.23	97.10	98.79	97.28	97.09
$ECFP_{6,4096}$	99.05	97.53	97.16	98.89	97.40	97.14	98.75	97.03	96.84	98.55	97.06	96.88
$ECFP_{8,4096}$	98.98	97.28	97.01	98.74	97.28	97.10	98.68	97.08	96.92	98.44	97.08	96.83
$ECFP_{10,4096}$	98.89	97.16	96.95	98.64	97.19	97.02	98.64	96.83	96.74	98.39	97.00	96.79
$ECFP_{6,8192}$	99.31	97.93	97.76	99.31	97.98	97.79	99.19	97.76	97.82	99.12	97.73	97.62
$ECFP_{6,16384}$	99.45	98.33	98.17	99.41	98.12	98.06	99.38	98.15	97.96	99.41	98.17	98.09
$ECFP_{6,32768}$	99.55	98.39	98.23	99.51	98.33	98.26	99.38	98.19	98.12	99.42	98.17	98.12

Analysis CDDD-space vs. ECFP-space

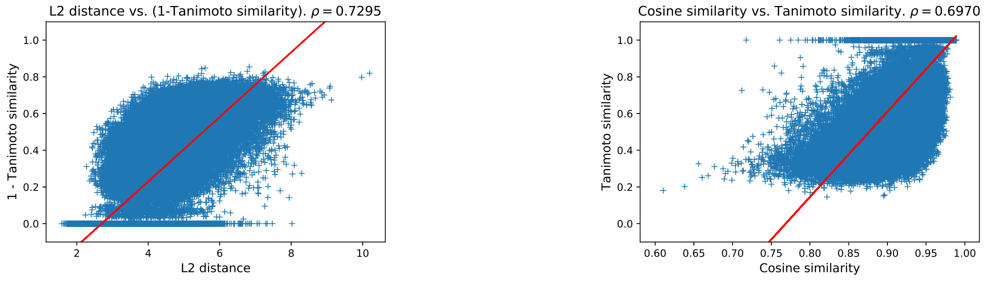

(a) (b)

Figure 2: Dependency between Euclidean (L2) distance and (1 – Tanimoto similarity) as well as Cosine similarity and Tanimoto similarity.

The $ECFP_{6,4096}$-count cluster-split model reports a reconstruction accuracy of 41.02% and mean Tanimoto similarity of 72.58% on the validation dataset (112, 332 samples). To illustrate the dependency between *CDDD*- and ECFP-space for the predicted deduced molecular structures, we computed the Euclidean distance and the Cosine similarity between predicted and true from the validation set. The dependency between Cosine similarity and Euclidean distance against Tanimoto similarity is shown in Figure 2. Since

we formulated the reverse-engineering task as machine learning problem of predicting a close sample, if not the correct sample, during training we aim to obtain a model f_θ, that minimizes the empirical loss function on the training set. Since the empirical loss function contains the deviance d, see Equation (1), the Euclidean distance is implicitly minimized as well. Figure 2a displays the positive correlation (pearson correlation coefficient of 0.7295) between L_2-distance and (1−Tanimoto similarity). As the Euclidean distance increases, the (1−Tanimoto similarity) increases. Interpreting the Euclidean distance and its magnitude in a high-dimensional space is difficult and not straightforward. The Cosine similarity benefits from its property being bounded within −1 and 1. Figure 2b shows the positive correlation (pearson correlation coefficient of 0.6970) between Cosine similarity and Tanimoto similarity. The red lines in Figure 2a and 2b display the linear functions, when regressing the y-axis on the x-axis, indicating the positive trend as well for both plots.

Analysis of hash collision

The classical ECFP is an *unfolded* fingerprint with no pre-defined size and its length depends on the input molecular structure. Since the ECFP algorithm iteratively uses a hash function that maps a list of atom environments (represented as integers) to a new atom environment $i \in 2^{32}$ and concatenates the result with the earlier list, the components of the final fingerprint can be large integers due to the target space of the hash function. To obtain *folded* binary or count-vectors from the unfolded fingerprint, the integer entries act as identifier for presence/counts and non-presence in the corresponding binary/count fingerprint. For example, consider a structure where the ECFP algorithm returns an unfolded fingerprint $[10, 10, 80, 999, 999999]$. This leads to an unfolded binary ECFP of length 99999, where the entries $\{10, 80, 999, 999999\}$ are populated with 1 and 0 elsewhere. The unfolded count fingerprint would have the component-value of 2 for the 10-th position, 1 in position $\{80, 999, 999999\}$ and 0 elsewhere. Now the "unfolded" binary/count-fingerprints are still variable in length, namely determined by

the maximum value of the unfolded ECFP, i.e. in the earlier example 999999. Since machine learning algorithms mostly require a fixed length feature input, the unfolded binary/count fingerprints are folded into fixed length k. The folding operation is usually applied with the modulo operation, by modulo-diving the "on"-positions/keys with k. Applying that, the folded bit/count fingerprint has length k. Assume we set $k = 10$ such that our bit/count fingerprints have fixed length of 10. Since the unfolded fingerprint is $[10, 10, 80, 999, 999999]$, indicating i-th's entries being "on", we now obtain the entries $[0, 0, 0, 9, 9, 9]$ being "on". For the binary ECFP this would mean that entries $\{0, 9\}$ are populated with 1 and 0 elsewhere. The count ECFP would be populated with the entry 3 in the components $\{0, 9\}$. Folding the fingerprint has led to a fixed fingerprint where only 2 unique keys $\{0, 9\}$ are on, whereas the original unfolded fingerprint had 4 unique keys $\{10, 80, 999, 9999\}$. Therefore, some information is lost. Here, we define the collision degree c as the difference of the number original unique keys and number of new unique keys, i.e. $c = 4 - 2 = 2$. Note that a collision degree of $c = 0$ means, that **no** information is lost after folding ECFP. Increasing k reduces the chance of collision and therefore the information loss.

For our $ECFP_6$ configurations, we computed the unfolded $ECFP_6$ vectors for all compounds in our processed dataset, obtained the number of unique keys and subtracted these values with the number of unique keys for the folded ECFP6 vectors. Figure 3a shows the results for increasing size k. As the fingerprint size k increases, the collision degree of larger than 1 decreases (or in other words, the collision degree of $c = 0$ increases).

Since our studies also include the analyses on the Neuraldecipher performance on a fixed fingerprint length $k = 4096$ but varying bond diameter $d \in \{4, 6, 8, 10\}$, we also computed the collision degrees for each of the five ECFP datasets with varying bond diameters. The results are shown in Figure 3b.

Figure 3a illustrates that the collision degree of $c = 0$, i.e. no information loss due to the folding operation, is highest for the $ECFP_6$ that was folded into length 32768, followed

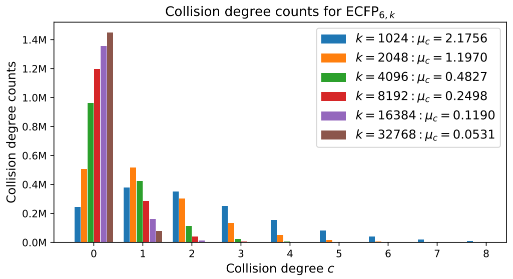

(a) Hash collision analysis for fixed $d = 6$ and increasing k

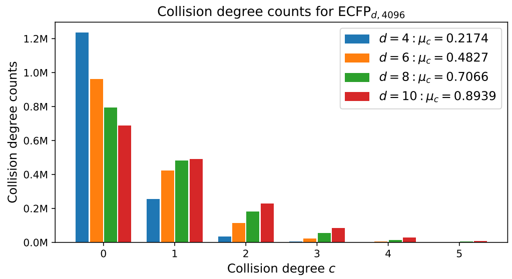

(b) Hash collision analysis for fixed $k = 4096$ and increasing d

Figure 3: Hash collision analysis for varying fingerprint length k and bond diameter d.

by 16384. The larger the fingerprint size, the smaller the average collision degree μ_c is for each setting. A higher average collision degree μ_c corresponds to more information loss.

When fixing the fingerprint length to $k = 4096$ and increasing the bond diameter d, we observe that the information loss also increases (see increasing average mean collision μ_c for increasing bond diamter d in Figure 3b). Since the unfolded $\text{ECFP}_{d'}$ with higher bond diameter $d' > d$ is a superset of the unfolded ECFP_d, the number of unique keys for the $\text{ECFP}_{d'}$ has *at least* the value of the number of unique keys for the ECFP_d. Since the two ECFPs are folded onto the same fixed length of $k = 4096$, it is natural that the ECFP with higher bond diameter suffers from more information loss. This information loss is shown in the higher number of counts for collisions degrees larger than 1, i.e. counts for $c \geq 1$.

Comparison Neuraldecipher trained on ECFP$_{6,4096}$-count vectors random split

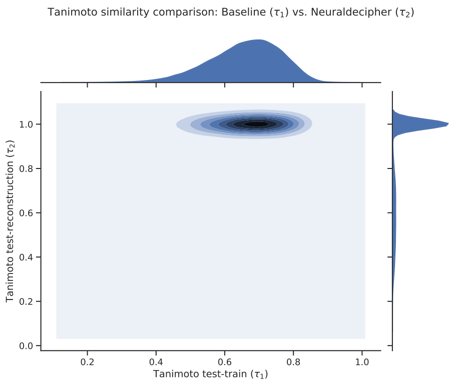

(a)

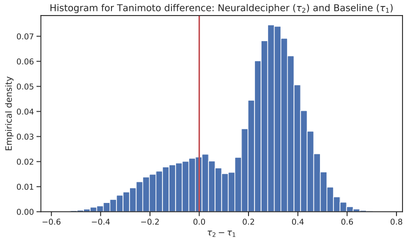

(b)

Figure 4: Comparison between the Neuraldecipher and Baseline model wrt. the Tanimoto similarity when trained on random split.

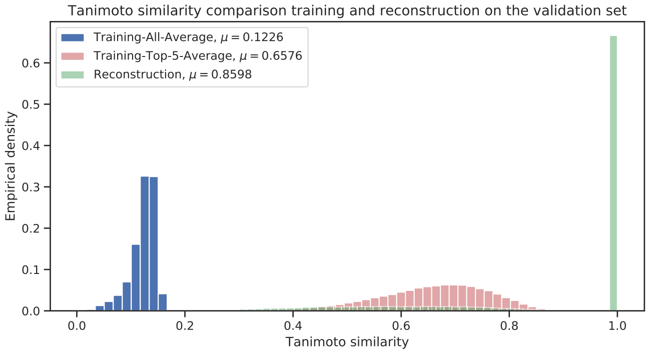

Figure 5: Histogram plot for the Tanimoto similarity between true SMILES representations and retrieved SMILES representation from the average training (blue), baseline model (red) and our reconstruction (green) on the validation set (112K samples) from the random split.

References

(1) Liaw, R.; Liang, E.; Nishihara, R.; Moritz, P.; Gonzalez, J. E.; Stoica, I. Tune: A Research Platform for Distributed Model Selection and Training. *arXiv preprint arXiv:1807.05118* **2018**,
